# Thiopental Inhibits Global Protein Synthesis by Repression of Eukaryotic Elongation Factor 2 and Protects from Hypoxic Neuronal Cell Death

**DOI:** 10.1371/journal.pone.0077258

**Published:** 2013-10-22

**Authors:** Christian I. Schwer, Cornelius Lehane, Timo Guelzow, Simone Zenker, Karl M. Strosing, Sashko Spassov, Anika Erxleben, Bernd Heimrich, Hartmut Buerkle, Matjaz Humar

**Affiliations:** 1 Department of Anesthesiology and Critical Care Medicine, University Medical Center Freiburg, Freiburg, Germany; 2 Department of General Neurosurgery, Cellular Neurophysiology, University Medical Center Freiburg, Freiburg, Germany; 3 Pharmaceutical Bioinformatics, Institute of Pharmaceutical Sciences, University of Freiburg, Freiburg, Germany; 4 Department of Anatomy and Cell Biology, University of Freiburg, Freiburg, Germany; University of Pecs Medical School, Hungary

## Abstract

Ischemic and traumatic brain injury is associated with increased risk for death and disability. The inhibition of penumbral tissue damage has been recognized as a target for therapeutic intervention, because cellular injury evolves progressively upon ATP-depletion and loss of ion homeostasis. In patients, thiopental is used to treat refractory intracranial hypertension by reducing intracranial pressure and cerebral metabolic demands; however, therapeutic benefits of thiopental-treatment are controversially discussed. In the present study we identified fundamental neuroprotective molecular mechanisms mediated by thiopental. Here we show that thiopental inhibits global protein synthesis, which preserves the intracellular energy metabolite content in oxygen-deprived human neuronal SK-N-SH cells or primary mouse cortical neurons and thus ameliorates hypoxic cell damage. Sensitivity to hypoxic damage was restored by pharmacologic repression of eukaryotic elongation factor 2 kinase. Translational inhibition was mediated by calcium influx, activation of the AMP-activated protein kinase, and inhibitory phosphorylation of eukaryotic elongation factor 2. Our results explain the reduction of cerebral metabolic demands during thiopental treatment. Cycloheximide also protected neurons from hypoxic cell death, indicating that translational inhibitors may generally reduce secondary brain injury. In conclusion our study demonstrates that therapeutic inhibition of global protein synthesis protects neurons from hypoxic damage by preserving energy balance in oxygen-deprived cells. Molecular evidence for thiopental-mediated neuroprotection favours a positive clinical evaluation of barbiturate treatment. The chemical structure of thiopental could represent a pharmacologically relevant scaffold for the development of new organ-protective compounds to ameliorate tissue damage when oxygen availability is limited.

## Introduction

Traumatic brain injury and cerebral infarction initiate deleterious events in the penumbra that exacerbate the initial injury [1], [2]. Cell death occurs when ATP production fails to maintain the energy supply for ionic and osmotic equilibrium [2], [3]. A rapid loss of high-energy phosphate compounds due to reduced blood flow or hypoxia results in a failure of ion-motive ATPases, membrane depolarization, excitotoxic glutamate release, and uncontrolled calcium influx, culminating in cell swelling, hydrolysis of proteins, inflammation, and cell death [3]–[7]. Limiting these deleterious responses might provide an adequate protection against ischemic injury and neuronal tissue damage.

Maintenance of ion homeostasis by ion-motive ATPases and protein synthesis are dominant energy-consuming processes of the cells [8], [9]. Depression of protein synthesis under conditions of insufficient oxygen and nutrient supply may result in substantial bioenergetic savings. Reallocation of cellular energy to vital mechanisms such as restoration of neuronal membrane potential or cellular repair may become critical for survival when ATP supply or availability of NAD+ is limited [9]–[11]. Inhibition of protein synthesis during ischemia may also prevent translation of inducible nitric oxide synthase (iNOS), cyclooxygenase-2 (COX-2), or matrix metalloproteinases (MMPs), that have been associated with peroxynitrite dependent nitration and oxidation of proteins or DNA, lipid peroxidation, inhibition of mitochondrial respiration, inflammation, and increased intracranial pressure or even haemorrhage due to blood-brain barrier leakage [4]–[6].

Protein synthesis depends on initiation and elongation factors whose activity is tightly regulated by posttranslational modification [12], [13]. Eukaryotic elongation factor 2 (eEF2) catalyzes the translocation of peptidyl-tRNA from the A site to the P site on the ribosome [12]. Phosphorylation of eEF2 at Thr56 by eEF2 kinase (eEF2K) impairs interaction of eEF2 with the ribosome [12], [14] and is sufficient for the inhibition of mRNA translation [15]. Phosphorylation of eEF2 at Ser595 by cyclin dependent kinase 2 facilitates Thr56 phosphorylation, probably by recruiting eEF2K to eEF2 [16]. eEF2K is a calcium/calmodulin dependent enzyme [13], [17], but it can independently be activated by cAMP-dependent protein kinase (PKA) [13], [18] or AMP-dependent protein kinase (AMPK) [13], [19]. Activation of eEF2K promotes cell survival, reduces hypoxic injury and regulates autophagy in response to nutrient deprivation [20]–[22]. Upon increased intracellular AMP/ATP ratios, AMPK induces ATP-generating catabolic pathways and simultaneous inhibits ATP-consuming pathways, thus regulating energy homeostasis [23]. Pathways, regulated by AMPK reduce ischemic cell damage [24], [25], inflammation [26], hypertrophy [27], plaque formation in Alzheimer’s disease [28], [29], or structural remodelling [30], and promote neurogenesis [31], angiogenesis [31], and blood flow [31]–[34].

The Brain Trauma Foundation Guidelines recommend high-dose thiopental treatment of patients with severe brain injury who present with refractory intracranial hypertension. This practice is the only second-level measure with class II evidence, demonstrating the ability of thiopental to reduce intracranial pressure [35]. However, a beneficial effect on neurological outcome is unproven and a critically discussed issue, mainly because of severe medical complications [36]. Although thiopental has been associated with inhibition of neuronal apoptosis [37], reduced excitotoxicity [38], [39], radical scavenging [40]–[42], and the induction of cytoprotective heat shock proteins [43], these experimental studies do not sufficiently explain major neuroprotective physiological observations such as decreased cerebral metabolism and reduced oxygen demand [44], [45].

Because cerebral metabolism and translation are closely intertwined, the aim of the present study was to examine thiopental-mediated effects on global protein synthesis, high-energy phosphate metabolism, and its impact on neuronal damage following oxygen deprivation.

## Materials and Methods

### Neuronal Cultures and Treatment with Chemicals

The human neuronal cell line SK-N-SH was purchased from the American Tissue Culture Collection (LGC Standards, Wesel, Germany) and maintained in Eagle’s minimum essential medium, supplemented with 1 mM sodium pyruvate, 2 mM Glutamax, 1500 mg/l sodium bicarbonate, 100 IU streptomycin, 100 IU penicillin, and 10% fetal calf serum (all from Life Technologies, Carlsbad, CA). Primary cortical neurons were dissected from C57/BL6 mouse neonates (P0–P2) as described [46]. Briefly, cortices were collected in cold modified Eagle’s medium supplemented with 2 mM Glutamax, cut with scissors to small pieces, and trypsinized in 0.25% trypsin for 10 min at 37°C. Then samples were dissociated in HBSS containing 10 mM HEPES-KOH (pH 7.9), 3 mg/ml bovine serum albumin, 12 mM MgSO_4_, 0.025% DNase I, and 0.4 mg/ml trypsin inhibitor from soybean (Applicem, Darmstadt, Germany) and triturated for 10–15 times using sterile glass Pasteur pipettes. After centrifugation at 100 g for 5 min, dissociated cells were collected from the supernatant. Neurons were enriched by a second centrifugation at 800 g for 5 min and seeded on Poly-d-Lysine coated culture plates in Dulbecco’s modified Eagle’s medium supplemented with 10% fetal calf serum and 2 mM Glutamax (Life Technologies) at 37°C, 8% CO_2_. After 3 h, the medium was changed to Neurobasal-A containing 2% B27-supplement and 1 mM Glutamax (Life Technologies).

Cells were treated with thiopental (Altana, Wesel, Germany), thapsigargin (Calbiochem, San Diego, CA), forskolin (Calbiochem), cycloheximide (Sigma-Aldrich, St. Louis, MO), and actinomycin D (Sigma-Aldrich) as indicated. The eEF2K inhibitor A484954, the calmodulin inhibitor camstatin, and the cAMP antagonist cAMPS-Rp were obtained from Tocris Bioscience (Bristol, UK). The AMPK inhibitor compound c (dorsomorphin dihydrochloride) was purchased from Calbiochem.

### Immunoblotting

Immunoblotting was performed using whole cell lysates as described previously [37]. The primary antibodies raised against eEF2, phospho-eEF2(Thr56), AMPK, and phospho-AMPK(Thr172) were obtained from Cell Signaling Technology (Danvers, MA). Protein bands were visualized using horseradish-peroxidase conjugated anti-rabbit IgG and enhanced chemiluminescence reagents (GE Healthcare, Little Calfont, UK).

### Metabolic Labeling

Cultures were incubated two times for 20 min in methionine free Eagle’s minimum essential medium (MP Biomedicals, Solon, OH) to deplete internal methionine pools before cells were pulse labeled with 200 µCi/ml of [^35^S]methionine (PerkinElmer, Rodgau, Germany) for 2 h and lysed in SDS-sample buffer (50 mM Tris-HCl/pH 6.8, 100 mM dithiothretiol, 2% sodiumdodecylsulfate, 10% glycerol, 0.1% bromphenol blue). Proteins were denatured by boiling for 5 min and separated by 10% SDS-PAGE. To demonstrate loading of equal amounts of protein, gels were fixed in 25% isopropanol and 10% acetic acid for 30 min, stained in 10% acetic acid containing 0.006% Coomassie G250 for 2 h, destained in 10% acetic acid for 6 h and dried on a Whatman 3 MM paper for 2 h at 80°C. [^35^S]methionine incorporation was visualized by exposure to x-ray films (GE Healthcare).

### Measurement of Intracellular Calcium

Intracellular cytoplasmatic calcium measurements were performed by fura-2 staining of SK-N-SH cells. Cultures were starved for 15 h, treated with thiopental or thapsigargin in calcium-free or calcium containing RPMI-medium (Genaxxon Bioscience, Ulm, Germany) and loaded with 2 µM fura-2 acetoxymethyl esther (Life Technologies) for the last 30 min of the experiment at 37°C. Fluorescence was excited at 340 nm and emission was recorded at 510 nm by a spectrofluorophotometer (SpectraMax® GeminiXS, Molecular Devices, Sunnyvale, CA).

### Measurement of Intracellular cAMP

Cells were plated in 96-well plates, starved over night at 80–90% confluence and incubated with thiopental or forskolin for 15 min to 6 h. Cells were then lysed in 0.1 M HCl for 10 min with moderate shaking at room temperature. The cellular cAMP content was determined using the competitive cAMP Parameter Assay Kit (R&D Systems, Wiesbaden-Nordenstadt, Germany) according to the manufacturer’s instructions. All values were normalized to internal standards and measured using a SpectraMax® Plus384 plate reader at 450 nm (Molecular Devices).

### Neuronal Cell Damage

Hypoxia-mediated neuronal cell death was induced by moving SK-N-SH cells or primary cortical neurons to freshly prepared growth medium that has been pre-equilibrated in a hypoxic atmosphere for 15 h (5% CO_2_, 95% N_2_) before placing them in a controlled atmosphere of 5% CO_2_, 95% N_2_ at 37°C for 72 h. Control cells were cultured in 5% CO_2_, 21% O_2_, and 74% N_2_ (normoxia).

Neuronal cell damage was analyzed by measurement of lactate dehydrogenase (LDH) in cell culture supernatants using the Cytotoxicity Detection Kit (Roche Applied Science, Mannheim, Germany) according to the manufacturer’s description. In brief, cell culture supernatants were taken 24–72 h after induction of hypoxia, added to LDH catalyst and dye solution, and incubated for 30 min at 25°C. The absorbance at 490 nm was measured in a spectrophotometer (SpectraMax® Plus384, Molecular Devices) with a reference wavelength of 690 nm. LDH values corresponding to 100% neuronal death were established by addition of 2% Triton-X 100 to untreated control cells (total cell lysis). Results were expressed as a percentage of neuronal death compared to total cell lysis.

### Caspase Activity Assay

Cells were lysed in 10 mM HEPES-KOH (pH 7.9), 350 mM NaCl, 1% Nonidet P-40, 1 mM MgCl_2_, 0.5 mM EDTA, 0.1 mM EGTA, 5 mM dithiotreitol, 2.5 mM phenylmethylsulfonyl fluoride, and 20 µg/ml aprotinin. Extracts were diluted 1∶10 in 100 mM HEPES-KOH (pH 7.5), containing 2 mM dithiotreitol and 60 µM of the fluorogenic caspase-3 substrate acetyl-DEVD-7-amino-4-methylcoumarin (Enzo Life Sciences, Loerrach, Germany). Caspase-3 like activity was determined in a Gemini XS plate reader at 340/460 nm (Molecular Devices) for 30 min at 27°C. Values were normalized to protein content and expressed as relative light units (RLU) per min.

### Measurement of Intracellular ATP

Intracellular ATP was measured by luciferase driven bioluminescence using the ATP Assay Kit (Calbiochem) according to the manufacturer’s description. Briefly, SK-N-SH cells or primary cortical neurons were cultured at 80–90% confluence in a controlled atmosphere of 5% CO_2_, 95% N_2_ in the presence or absence of thiopental or cycloheximide at 37°C for 12–72 h in 6-well plates. Control cells were cultured under normoxic conditions. Then cells were placed in 250 µl ice-cold ATP-releasing agent (20 mM Tris-HCl pH 7.4, 150 mM NaCl, 0.1% sodium dodecyl sulfate, 0.5% sodium deoxycholate, 1% Nonidet P-40, 100 µg/ml phenylmethylsulfonyl fluoride, 1 mM sodium orthovanadate, 70 µg/ml of aprotinin) and lysis was allowed to proceed by repeated freeze/thaw cycles. Cell lysates were diluted 1∶5 in 40 µl nucleotide releasing buffer and luciferase activity was determined by a luminometer (Microluminat Plus LB 96P; Berthold Technologies, Bad Wildbad, Germany) by automated injection of 50 µl luciferin–luciferase mixture from the ATP Assay Kit. Values were normalized to cellular protein concentrations.

### Statistical Analysis

Results are expressed as means ± standard deviations for the indicated number of separate experiments. Statistical differences between experimental groups were determined by performing one-way ANOVA followed by the Dunnett’s multiple comparisons test. Data describing cAMP-levels, LDH release, or ATP content were evaluated by two-way ANOVA, followed by the Bonferroni’s *post hoc* test. Differences between groups were considered to be significant at p<0.05. Statistical analyzes were carried out using the Prism software package (GraphPad Software Inc., La Jolla, CA, USA).

## Results

### Thiopental Inhibits Global Protein Synthesis by Induction of eEF2 Phosphorylation

**Figure 1:**
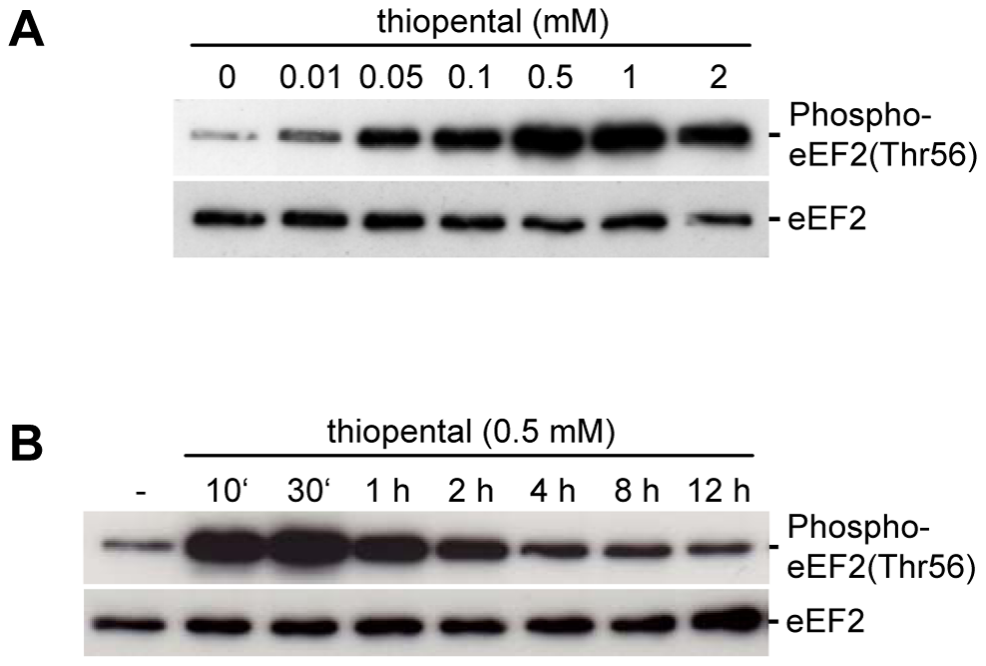
Thiopental induces eEF2 phosphorylation. SK-N-SH cells were treated with 10 µM –2 mM thiopental for 6 h (A) or with 0.5 mM thiopental for 10 min to 12 h (B) and analyzed by immunoblotting with an anti-human phospho-eEF2 threonine 56 antibody (upper blots) or an eEF2 antibody that detects endogenous levels of eEF2 independently of phosphorylation (lower blots). Data are representative of four independent experiments.

Thiopental is used as a neuroprotective agent in patients with severe brain injury to decrease intracranial pressure, cerebral metabolism and the demand of cerebral oxygenation [44], [45]. Although thiopental-mediated physiological effects are well documented, the molecular mechanisms that contribute to neuroprotection are unclear. Cerebral metabolism is marked by translational activity, with elongation consuming the majority of cellular energy [12], [13]. Elongation is regulated by eEF2, a translocase that is tightly controlled by posttranslational modification and necessary for the movement of the mRNA along the ribosome [12], [13]. To test whether thiopental affects translation by modulating the elongation step during ribosomal protein synthesis, we treated human neuronal SK-N-SH cells with 0.01–2 mM thiopental which is within the range of concentrations observed in the plasma or tissue of patients receiving this drug [47]–[49]. Our results clearly indicate that phosphorylation of eEF2 at Thr56 is induced by thiopental (Figure 1). This inhibitory modification was already observed in the presence of 10 µM thiopental and was most pronounced at 0.5 mM or higher. Phosphorylation of eEF2 was induced within 10 min and persisted for at least 12 h. The concentrations of thiopental used in our experiments resulted in no apparent toxic effects as lactate dehydrogenase levels in cell culture supernatants were not increased when SK-N-SH cells were cultured in the presence or absence of thiopental for 72 h (data not shown). The total amount of eEF2 was unchanged by exposure to thiopental (Figure 1).

**Figure 2:**
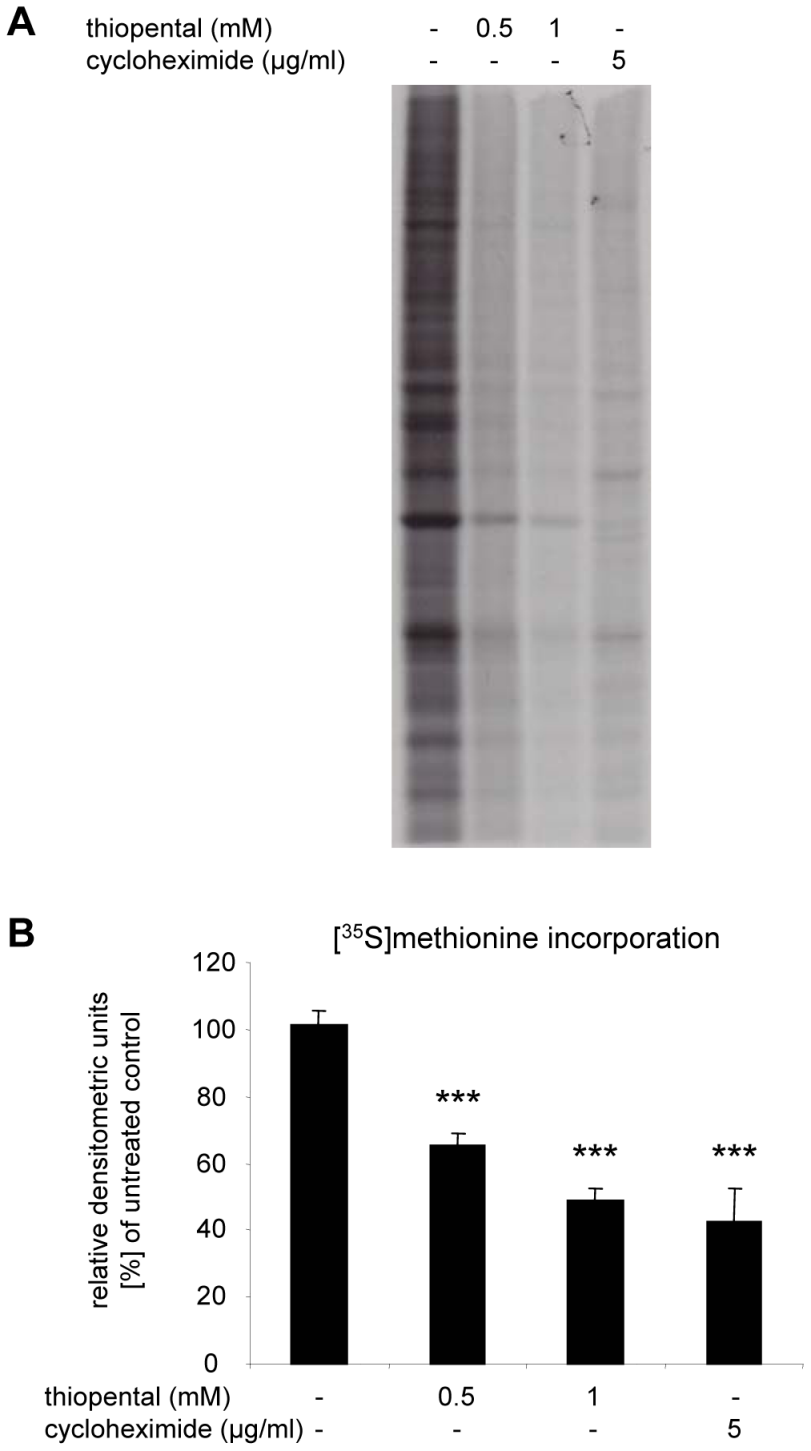
Thiopental inhibits global protein synthesis. SK-N-SH cells were left untreated or were exposed to 0.5 mM thiopental, 1 mM thiopental, or 5 µg/ml cycloheximide for 6 h and then pulsed with [^35^S]methionine for an additional 2 h. Cell lysates were separated by SDS-PAGE. The amounts of newly synthesized proteins were detected by autoradiography on dried electrophoresis gels (A) and quantified by densitometry (B). Values represent the means ± standard deviations. Statistical differences between experimental groups were determined by performing one-way ANOVA followed by the Dunnett’s multiple comparisons test. ***, p<0.001 versus untreated control group (first bar). The results shown are representative of three independent experiments.

Phosphorylation of eEF2 at Thr56 is sufficient to inhibit global protein synthesis [14], [15]. To determine whether thiopental altered the basal rate of protein synthesis in neuronal SK-N-SH cells, neurons were metabolically labeled with [^35^S]methionine, and the extend of synthesized nascent polypeptides was visualized by SDS-PAGE and autoradiography. To ensure that incorporation of [^35^S]methionine was directly proportional to the length of labeling time, SK-N-SH cells were labeled for 20 min to 6 h in preliminary experiments. [^35^S]methionine incorporation into proteins was linear within this time period (data not shown). Consequently, cells were pulse-labeled with [^35^S]methionine for 2 h in the following experiments. Our results depicted in Figure 2 clearly demonstrate, that thiopental significantly reduces the basal rate of protein synthesis in a dose dependent manner. Treatment of cells for 6 h with cycloheximide, a known inhibitor of translation, also resulted in a marked decrease in protein synthesis (Figure 2). In contrast, total protein levels visualized by Coomassie G250 staining of gels before autoradiography were similar (data not shown). These findings indicate that thiopental does not selectively downregulate protein expression but instead exerts a global inhibitory effect on protein translation. The ability of thiopental to decrease global protein synthesis is consistent with the observed increase in eEF2 phosphorylation.

### Thiopental Elevates Cytoplasmatic Calcium Levels

**Figure 3:**
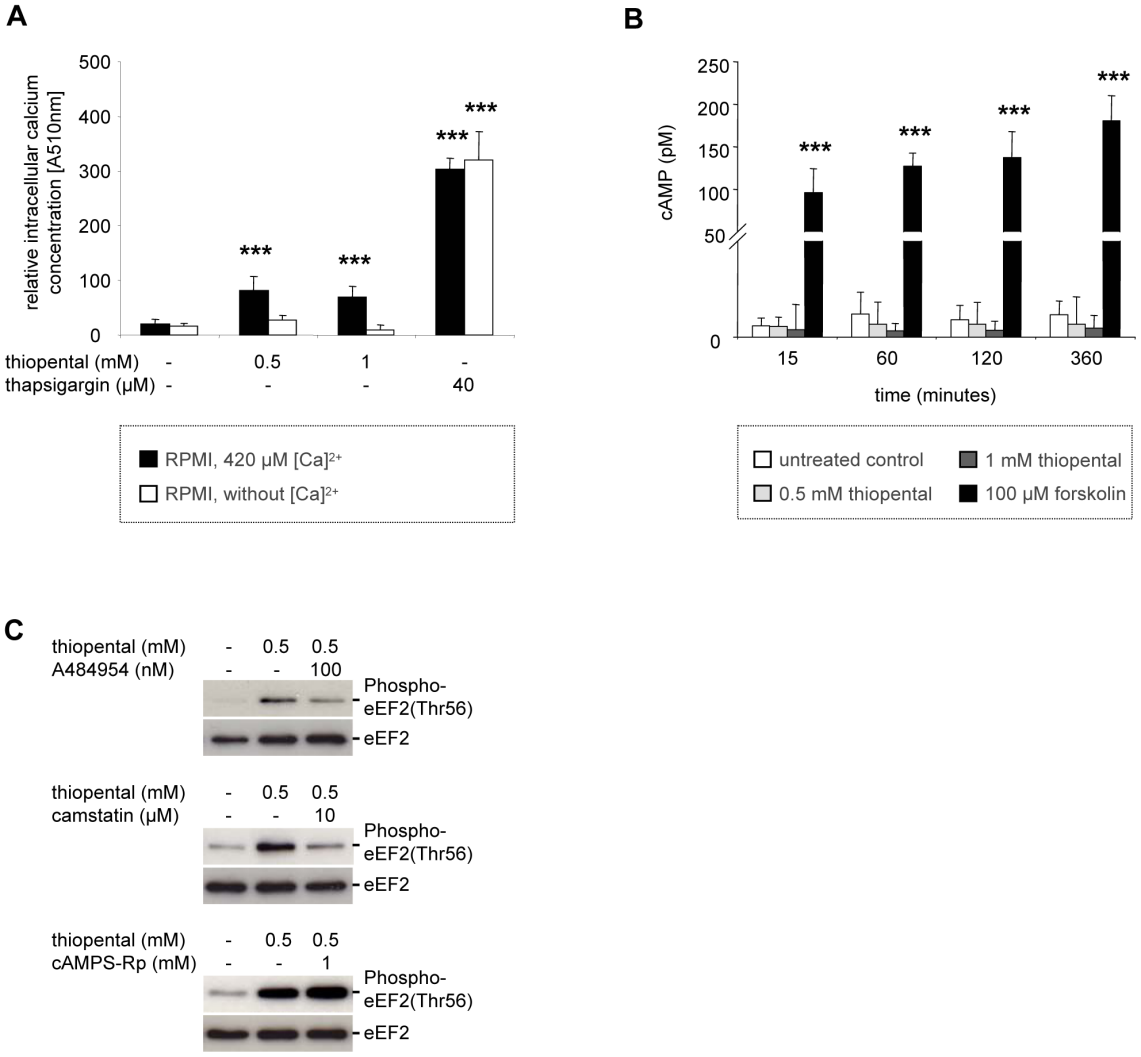
Thiopental increases intracellular calcium levels but not cAMP content. (A), SK-N-SH cells were left untreated or were incubated in the presence of 0.5 mM thiopental, 1 mM thiopental, or 40 µM thapsigargin in calcium containing (black bars) or calcium-free (white bars) RPMI medium for 1 h. For the last 30 min of the experiment cells were loaded with 2 µM fura-2 acetoxymethyl ester before intracellular calcium-complexes were excited at 340 nm and recorded at 510 nm. Values represent the means ± standard deviations. Experimental groups were statistically analyzed by performing one-way ANOVA followed by the Dunnett’s multiple comparisons test. ***, p<0.001 versus untreated control group, n = 3. In (B), SK-N-SH cells were left untreated or were incubated with 0.5 mM thiopental, 1 mM thiopental, or 100 µM forskulin for 15 min to 6 h before cell lysis. Cellular cAMP content was determined by a competitive cAMP immunoassay. Values represent the means ± standard deviations, n = 3. Experimental groups were statistically analyzed by performing two-way ANOVA followed by the Bonferroni’s *post hoc* test. Statistical differences versus untreated control cells are shown (***, p<0.001). In (C), immunoblots were performed using cells that were pretreated with 100 µM A484954, 10 µM camstatin, or 1 mM cAMPs-pR for 1 h before 0.5 mM thiopental were added for the last hour of the experiment. The immunoblots were analyzed with an anti-human phospho-eEF2 threonine 56 antibody or an anti-eEF2 antibody that detects endogenous levels of eEF2 independently of phosphorylation. Data are representative of three independent experiments.

eEF2 phosphorylation is regulated by the calcium and calmodulin-dependent kinase eEF2K [17]. To examine whether eEF2K is involved in regulating Thr56 phosphorylation of eEF2, we first analyzed the effect of thiopental on cytoplasmatic calcium content. Free intracellular calcium imaging was performed by labeling SK-N-SH cells with the membrane-permeable fluorometric calcium indicator Fura-2AM. As illustrated in Figure 3A, thiopental increased fura-2 fluorescence by 4 to 6-fold. This increase was dependent on the presence of extracellular calcium, suggesting an opening of plasma membrane gated calcium channels. In contrast, thapsigargin also elevated cytoplasmatic calcium levels in the absence of extracellular calcium, demonstrating a different mechanism of calcium mobilization. This observation is in agreement with previous reports, in which thapsigargin is described as an inhibitor of the sarcoplasmic and endoplasmic reticulum calcium ATPase that deregulates intracellular calcium homeostasis by blocking calcium uptake into intracellular storage compartments and depletion of these stores.

Several protein kinases regulate calcium-independent eEF2K activity [13]. The cAMP dependent protein kinase (PKA) stimulates eEF2K by site-specific phosphorylation [18]. To examine whether thiopental affects cAMP signaling, the amount of cAMP in thiopental treated SK-N-SH cells was determined by a competitive enzyme immunoassay. Independent studies described a dose-dependent inhibition of beta-adrenergic stimulated cAMP production in myocardial tissue by thiopental [50]. In contrast, we observed no significant changes in cytosolic cAMP in resting neuronal SK-N-SH cells during thiopental treatment (Figure 3B). This conflicting results might be due to the fact, that opposed to other groups adenylate cyclase was chemically not pre-activated in our experiments [50]. However, forskolin, a common activator of the cAMP/PKA/eEF2K/eEF2 pathway, dramatically increased cellular cAMP levels, demonstrating that adenylate cyclase may principally be induced in neuronal SK-N-SH cells (Figure 3B).

The significance of eEF2K, calcium-dependent calmodulin, and cAMP in eEF2 phosphorylation were additionally analyzed by the use of the eEF2K inhibitor A484954, the calmodulin antagonist camstatin, and the cAMP analog cAMPS-Rp. As shown in Figure 3C, incubation of neuronal SK-N-SH cells with A484954 or camstatin reduced thiopental-dependent eEF2 phosphorylation, whereas the cAMP antagonist cAMP-Rp had no effect. These experiments confirm that thiopental induced phosphorylation of eEF2 is mediated by calcium/calmodulin dependent eEF2K activation but not by the cAMP/PKA/eEF2K pathway.

### Thiopental Increases eEF2 Phosphorylation by an AMPK-dependent Mechanism

**Figure 4:**
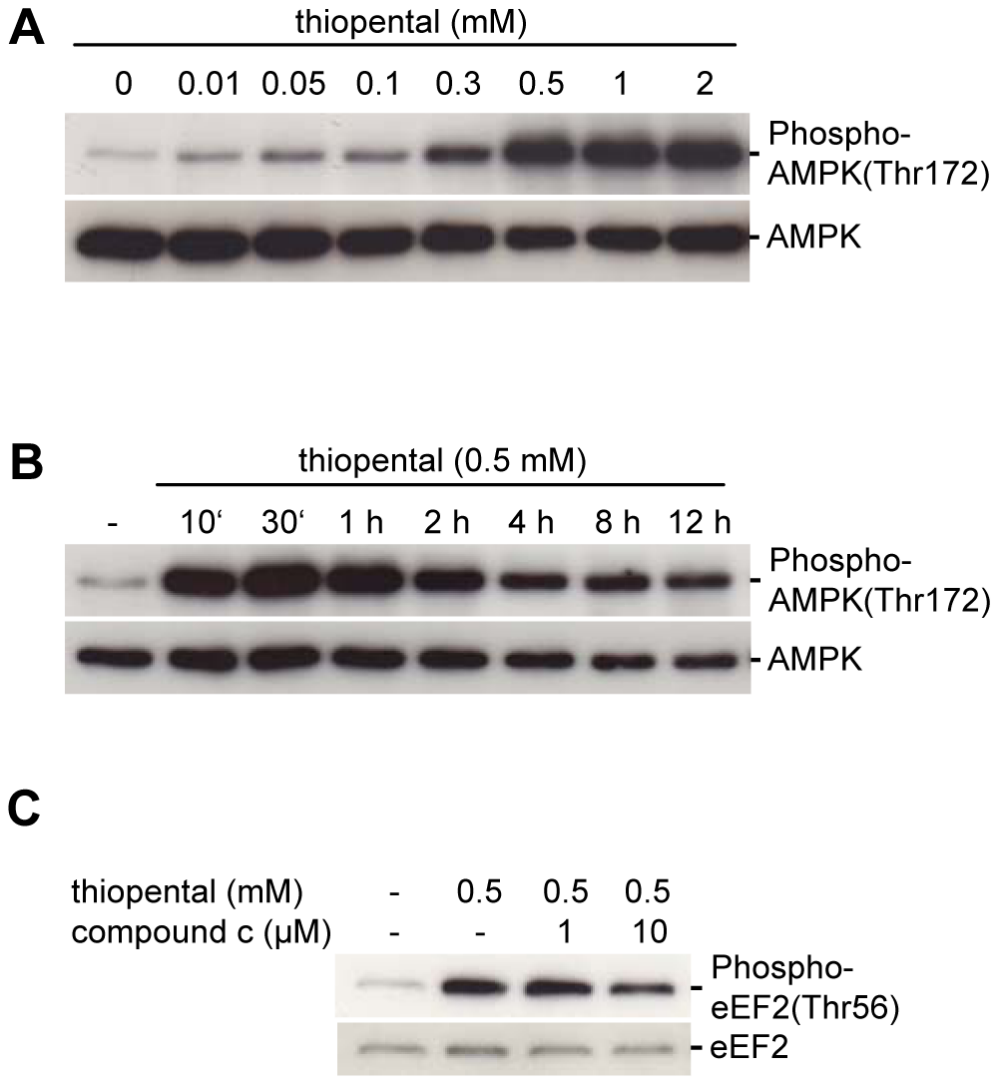
Thiopental induces AMPK autophosphorylation. Cells were treated with 10 µM –2 mM thiopental for 6 h (A) or with 0.5 mM thiopental for 10 min to 12 h (B) and analyzed by immunoblotting with an anti-human phospho-AMPK threonine 172 antibody (upper blots) or an AMPK antibody that detects endogenous levels of total AMPK independently of phosphorylation (lower blots). In (C), AMPK was inhibited by 1–10 µM compound c for 1 h before 0.5 mM thiopental was added for the last hour of the experiment. Phosphorylation of eEF2 at threonine 56 (upper blot) or endogenous levels of eEF2 independently of phosphorylation (lower blot) were quantified by immunoblotting. Data are representative of three independent experiments.

Barbiturates inhibit mitochondrial respiration [51]. Although effects of barbiturates on oxidative phosphorylation have not been recognized previously as physiologically relevant in neurons, we speculated that transiently elevated AMP/ATP ratios might lead to induction of AMPK, a kinase that can activate eEF2K through phosphorylation at Ser398 [19], and thus might be responsible for the inhibitory Thr56 phosphorylation of eEF2. As shown in Figure 4, exposure of neuronal SK-N-SH cells to thiopental resulted in a substantial increase in the levels of activated AMPK within 10 min that persisted for at least 12 h. The observed phosphorylation of AMPK at Thr172 in the activation loop is required for AMPK activation [52]. Furthermore, AMPK phosphorylation at Thr172 was dose-dependent, whereas the total amount of AMPK was unchanged by exposure to thiopental.

Next we investigated the importance of AMPK in eEF2K-dependent eEF2 phosphorylation. Because the eEF2K-Ser398 antibody is commercially not available, we directly analyzed eEF2 phosphorylation in the presence of the AMPK inhibitor compound c. Surprisingly, thiopental induced eEF2 phosphorylation was only partially reduced by 10 µM of compound c (Figure 4C), suggesting the presence of an additional dominant pathway that induces eEF2K-dependent eEF2 inactivation when AMPK is blocked. Indeed, in Figures 3A and 3C we demonstrated a rise in intracellular calcium and a calcium/calmodulin dependent phosphorylation of eEF2 that may be independent of AMPK [13]. Based on these results we postulate that thiopental mediated inactivation of eEF2 depends on induction of AMPK and calcium/calmodulin complexes.

### Thiopental Protects Neuronal SK-N-SH Cells from Hypoxic Cell Damage by Inhibition of Global Protein Synthesis

**Figure 5:**
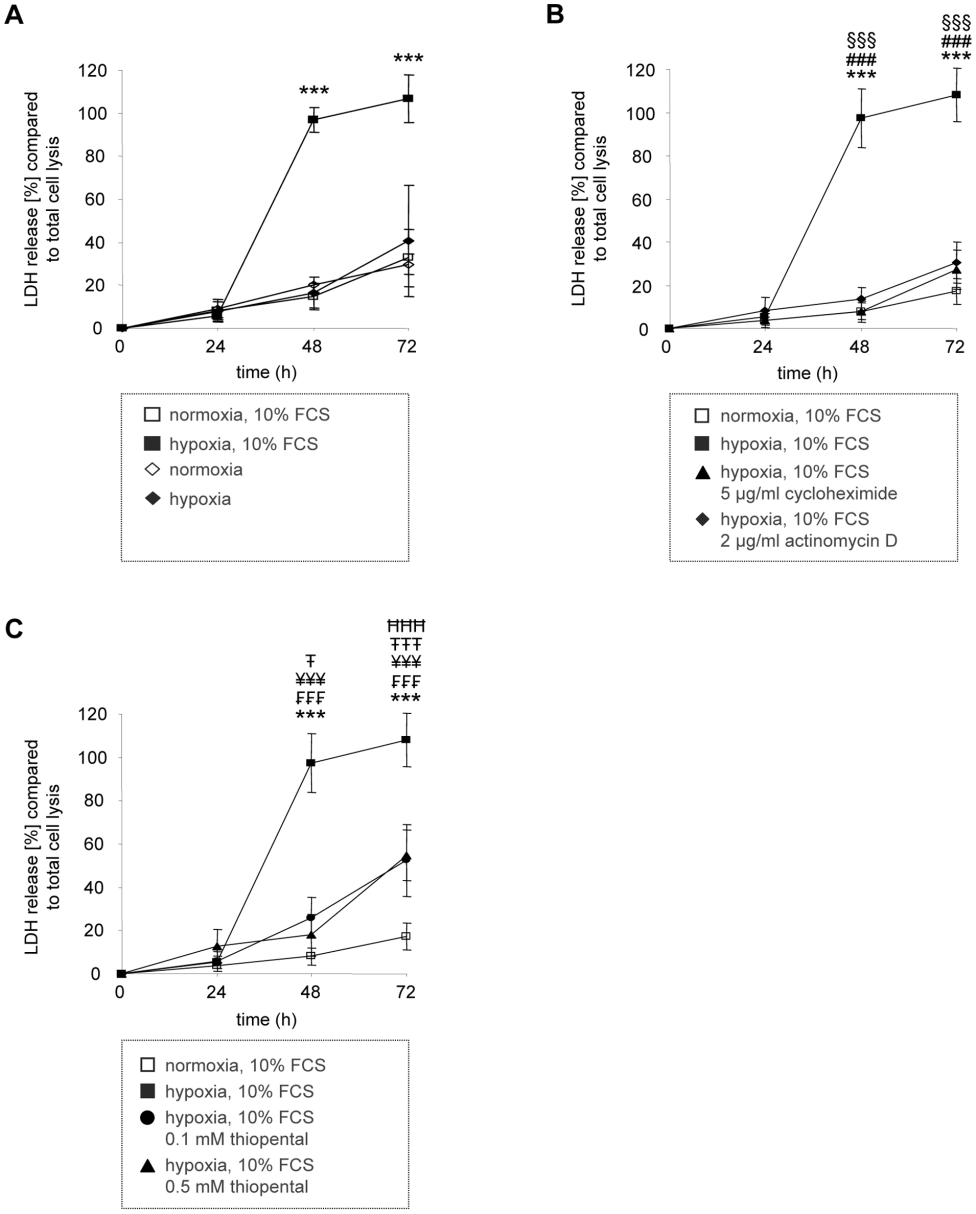
Inhibitors of protein synthesis reduce hypoxic neuronal damage. Cellular damage in human neuronal SK-N-SH cells was induced by oxygen deprivation (closed symbols) in an atmosphere containing 5% CO_2_, 95% N_2_ for 0–72 h and determined by an LDH release assay. Control cells were cultured in 5% CO_2_, 21% O_2_, and 74% N_2_ (open symbols). In (A), cellular damage was measured in the presence (squares) or absence (rhombi) of fetal calf serum. In (B), 5 µg/ml cycloheximide (closed triangles) or 2 µg/ml actinomycin D (closed rhombi) were added to the cells in serum containing growth medium (squares). In (C), 0.1 mM thiopental (closed circles) or 0.5 mM thiopental (closed triangles) were added to the cells in serum containing growth medium (squares). Values represent the mean ± standard deviations of four separate experiments. Experimental groups were statistically analyzed by performing two-way ANOVA followed by the Bonferroni’s *post hoc* test. Statistically significant differences within groups shown for (A) are: serum treated oxygen deprived SK-N-SH cells versus serum treated normoxic control cells (***, p<0.001). Statistically significant differences within serum treated groups shown for (B) are: normoxic control cells versus oxygen deprived SK-N-SH cells (***, p<0.001); and oxygen deprived SK-N-SH cells versus oxygen-deprived cells treated with 2 µg/ml actinomycin D (^§§§^, p<0.001) or versus oxygen-deprived cells treated with 5 µg/ml cycloheximide (^###^, p<0.001). Statistically significant differences within serum treated groups shown for (C) are: normoxic control cells versus oxygen deprived SK-N-SH cells (***, p<0.001), versus oxygen-deprived cells treated with 0.1 mM thiopental (^<$>\raster(60%)="rg1"<$>^, p<0.05; ^<$>\raster(60%)="rg1"<$><$>\raster(60%)="rg1"<$><$>\raster(60%)="rg1"<$>^, p<0.001) or versus oxygen-deprived cells treated with 0.5 mM thiopental (^<$>\raster(60%)="rg2"<$><$>\raster(60%)="rg2"<$><$>\raster(60%)="rg2"<$>^, p<0.001); and oxygen deprived SK-N-SH cells versus oxygen-deprived cells treated with 0.1 mM thiopental (^<$>\raster(60%)="rg3"<$><$>\raster(60%)="rg3"<$><$>\raster(60%)="rg3"<$>^, p<0.001) or versus oxygen-deprived cells treated with 0.5 mM thiopental (^¥¥¥^, p<0.001).

The penumbra, surrounding the primary necrotic core in brain injured patients, is composed of a rim of ischemic tissue potentially destined for infarction but not yet irreversibly injured [1], [2]. As hypoxic cell death is not imminent in this region it is a central target of acute therapies to salvage secondarily damaged ischemic cells. Our intention was to explore whether translational repressors such as thiopental reduce hypoxic cell damage and thus might be suitable to rescue brain tissue by limiting the volume of the penumbra. Therefore, neuronal SK-N-SH cells were subjected to oxygen deprivation and cellular damage was accessed by determination of intracellular lactate dehydrogenase in cell culture supernatants. Interestingly, we observed that hypoxia induced cell damage was most pronounced in serum activated neuronal SK-N-SH cells whereas serum starved cells seemed largely unaffected (Figure 5A). Significant hypoxic damage was first observed at 48 h, thus providing a sufficient time frame for pharmacological treatment to reduce hypoxic cell death. Delayed ischemic cell death is a well documented event in the penumbra [1], [2].

The observed difference in susceptibility to hypoxic neuronal cell death would imply that metabolically active cells are particularly vulnerable to oxygen deprivation, whereas metabolic inactive, resting cells are protected under these conditions. Therefore we speculated that inhibitors of protein synthesis might significantly reduce hypoxic cell damage. To examine whether a reduction in global protein synthesis prevents ischemic damage, we first used the translational inhibitor cycloheximide to test the viability of oxygen deprived neuronal cells in the presence of serum (Figure 5B). In agreement with our assumption, cycloheximide completely blocked hypoxic damage for 72 h. In the presence of cycloheximide the amount of lactate dehydrogenase was comparable in supernatants of normoxic and oxygen deprived cells. Likewise, the transcriptional repressor actinomycin D reduced hypoxic damage to a similar extent (Figure 5B). These experiments prove that inhibitors of global protein synthesis represent an effective means to protect neuronal cells from cell death by oxygen deprivation.

**Figure 6:**
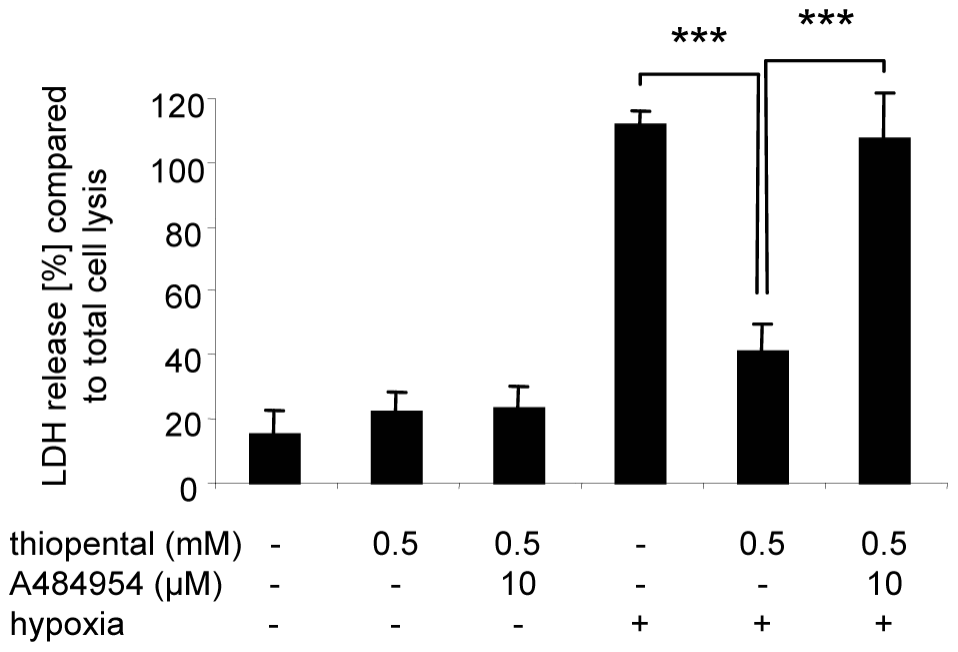
Inhibition of hypoxic damage by thiopental is mediated by eEF2K. SK-N-SH cells were cultured in the presence or absence of 10 µM A484954 for 1 h before addition of 0.1 mM thiopental and exposure to hypoxia. After 72 h cellular damage was determined by an LDH-release assay. Values represent the mean ± standard deviations of four independent experiments. Statistical evaluation of experimental groups was performed by one-way ANOVA followed by the Bonferroni’s *post hoc* test. The statistically significant difference of thiopental treated, oxygen-deprived SK-N-SH cells in the presence or absence of A484954 is shown (***, p<0.001).

In the following experiments we examined whether thiopental, that has previously been shown to inhibit global protein translation also mediates cytoprotection during hypoxia. As shown in Figure 5C, thiopental significantly reduced hypoxic cell damage in a time dependent manner. However the protective effect decreased after 48 h, probably due to a limited half life of thiopental which has been described as being between 6 and 46 h [53]. Indeed, neuroprotection by thiopental has been observed in the clinic after continuous high dose barbiturate coma [54]. Pharmacologic inhibition of eEF2K by A484954 restored sensitivity to hypoxic damage in thiopental-treated neuronal cells, demonstrating that eEF2K kinase activity is an essential event to confer resistance to cell death following oxygen deprivation (Figure 6).

**Figure 7:**
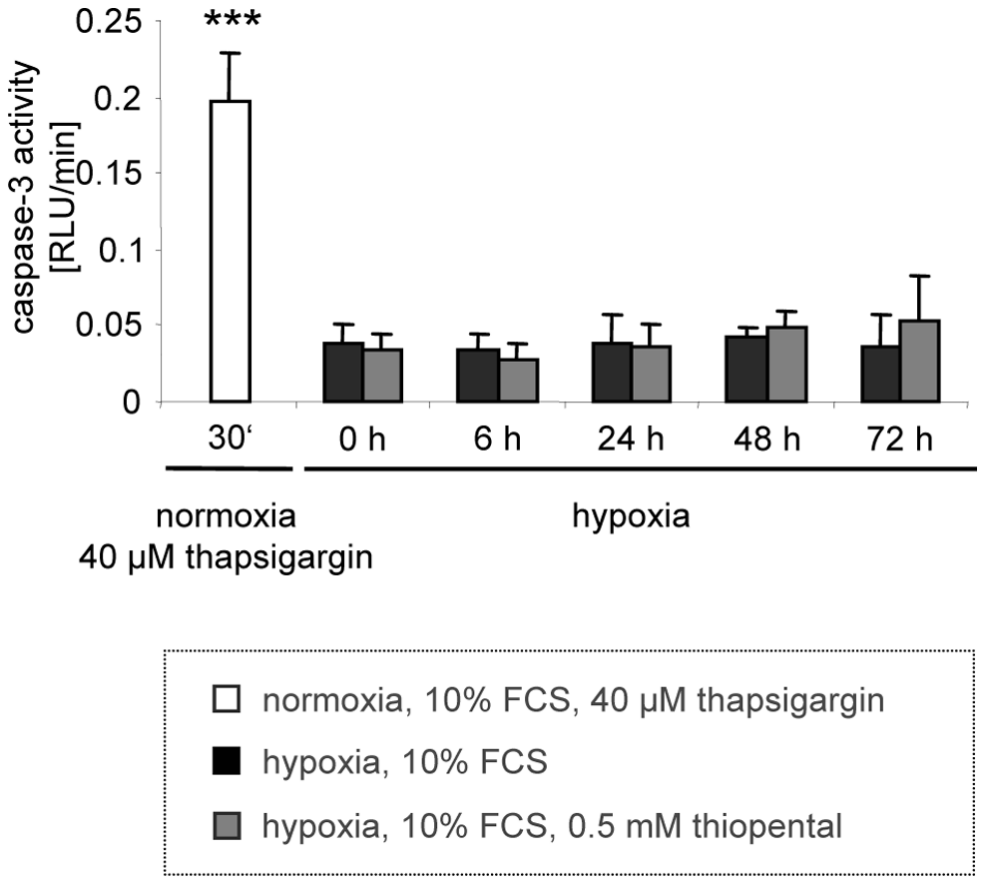
Hypoxic neuronal damage is independent of caspase-3. SK-N-SH cells were cultured in the presence (grey bars) or absence (black bars) of 0.5 mM thiopental without oxygen for 0–72 h before caspase-3 like activity was assessed by a fluorogenic caspase-3 activity assay. Values are expressed as relative light units (RLU)/min and were normalized to protein content. As a control, apoptosis was induced by 40 µM thapsigargin in normoxic SK-N-SH cells for 30 min (white bar). Statistic evaluation of experimental groups was performed by one way ANOVA followed by the Dunnett’s multiple comparisons test. ***, p<0.001 versus untreated control cells (black bar, 0 h hypoxia). The results shown are representative of three independent experiments.

Next we examined the execution of hypoxic neuronal cell death. Surprisingly we could neither demonstrate caspase-3 activity in oxygen deprived SK-N-SH cells (Figure 7), nor were we able to detect cleaved caspase-3 in extracts of these cells (data not shown). In contrast, thapsigargin induced a strong release of calcium (Figure 3A), significant caspase-3 activity (Figure 7), and cell death (data not shown). It is well accepted that disruption of calcium homeostasis might result in neuronal death [7]. However, based on our results caspase-3 dependent cell death is not involved in hypoxic neuronal cell damage.

In Figure 3A we have shown that thiopental induces an increase in cytoplasmatic calcium levels, which might be associated with neuronal cell death [7]. However, compared to thapsigargin, thiopental mediated changes of intracellular calcium levels were moderate and thus not sufficient to induce calcium-mediated apoptosis. In fact, thiopental treated oxygen-depleted cells were not able to induce caspase-3 (Figure 7). In contrast to the lethal increase of calcium induced by thapsigargin treatment, the moderate changes of calcium levels mediated by thiopental may be suitable for the activation of protective physiological processes such as activation of calcium/calmodulin dependent eEF2K and eEF2 phosphorylation.

### Inhibition of Global Protein Synthesis by Thiopental Preserves Intracellular ATP Content

**Figure 8:**
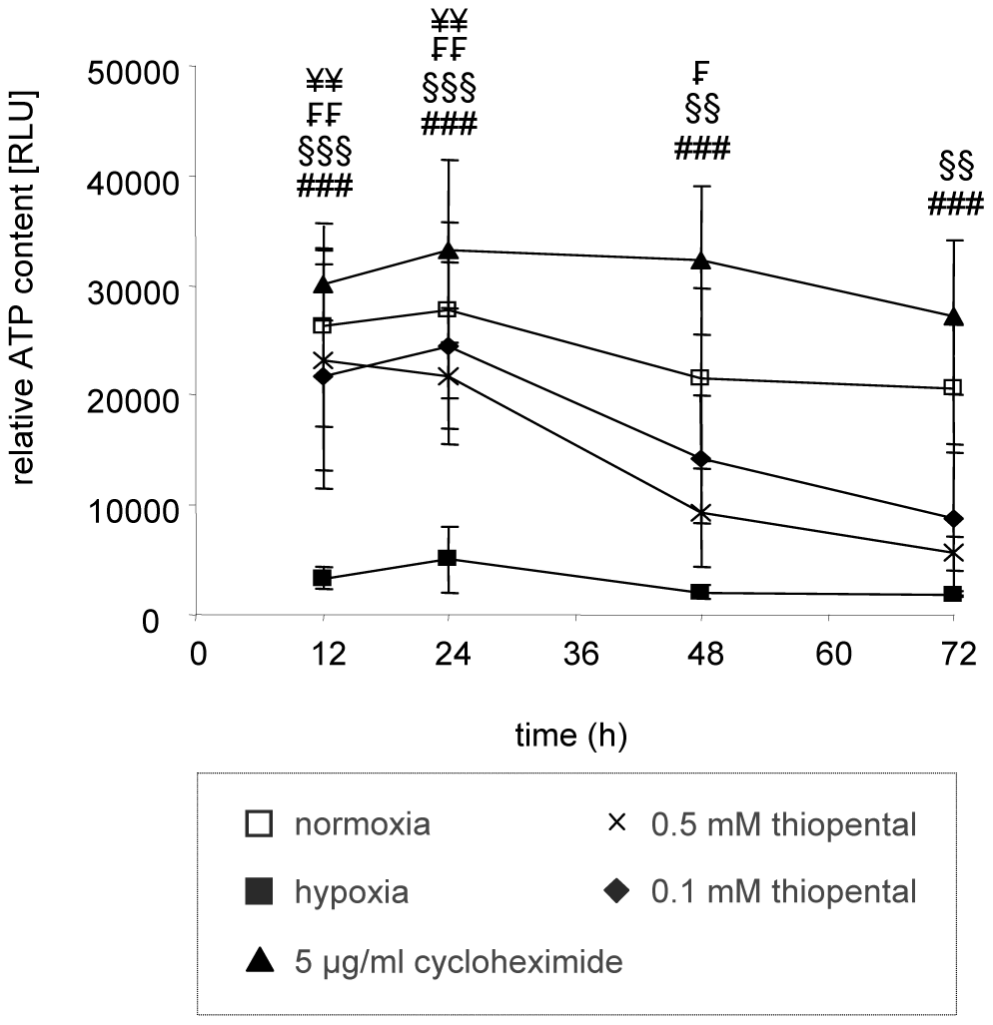
Inhibitors of protein synthesis preserve intracellular ATP-content during oxygen deprivation. Human neuronal SK-N-SH cells were cultured in an oxygen-free atmosphere for 12–72 h in the presence of 5 µg/ml cycloheximide (closed triangles), 0.1 mM thiopental (closed rhombi), 0.5 mM thiopental (asterisks), or left untreated (closed squares). Cells, cultured in a normoxic atmosphere (open squares) served as a control. ATP content of cells was measured in lysates by an ATP-driven luciferase assay. Determined relative light units (RLU) were normalized to protein content and represent the means ± standard deviations of three independent experiments. Experimental groups were statistically evaluated by performing two-way ANOVA followed by the Bonferroni’s *post hoc* test. Statistical differences of oxygen deprived SK-N-SH cells (closed squares) compared to oxygen-deprived cells treated with 5 µg/ml cycloheximide (^###^, p<0.001), 0.5 mM thiopental (^<$>\raster(60%)="rg3"<$>^, p<0.05; ^<$>\raster(60%)="rg3"<$><$>\raster(60%)="rg3"<$>^, p<0.01), 0.1 mM thiopental (^¥¥^, p<0.01), or untreated control cells (^§§^, p<0.01; ^§§§^, p<0.001) are shown.

mRNA translation is an energy-consuming process that depletes up to 60% of the ATP synthesized by the cells [8]. Small adjustments to the translation rate may spare sufficient ATP to sustain critical functions for ischemic cell survival and thus antagonize hypoxic cell damage [8], [9]. Therefore we analyzed whether protection from hypoxic cell damage by cycloheximide or thiopental is accompanied by a preservation of intracellular ATP content. Oxygen deprived neuronal SK-N-SH cells displayed a massive decrease in intracellular ATP that was already maximal after 12 h (Figure 8). Addition of cycloheximide prevented the reduction in levels of ATP. In fact, hypoxic cycloheximide treated cells consistently showed an increased ATP content compared to untreated normoxic cells. Treatment of oxygen deprived cells with the transcriptional repressor actinomycin D confirmed these observations (data not shown). Thiopental also conserved ATP levels in oxygen-deprived neurons, although ATP content progressively declined with duration of hypoxia (Figure 8). In contrast to cycloheximide and actinomycin D, ATP-levels in thiopental treated cells never exceeded ATP content of untreated cells. This is consistent with observations describing thiopental as a moderate inhibitor of mitochondrial respiration [51]. The observed progressive reduction of ATP-levels in thiopental treated hypoxic cells might be due to a time dependent loss in thiopental activity [53] and correlates with reduced protection from hypoxic damage (Figure 5C).

### Translational Control by Thiopental in Primary Cortical Neurons

**Figure 9:**
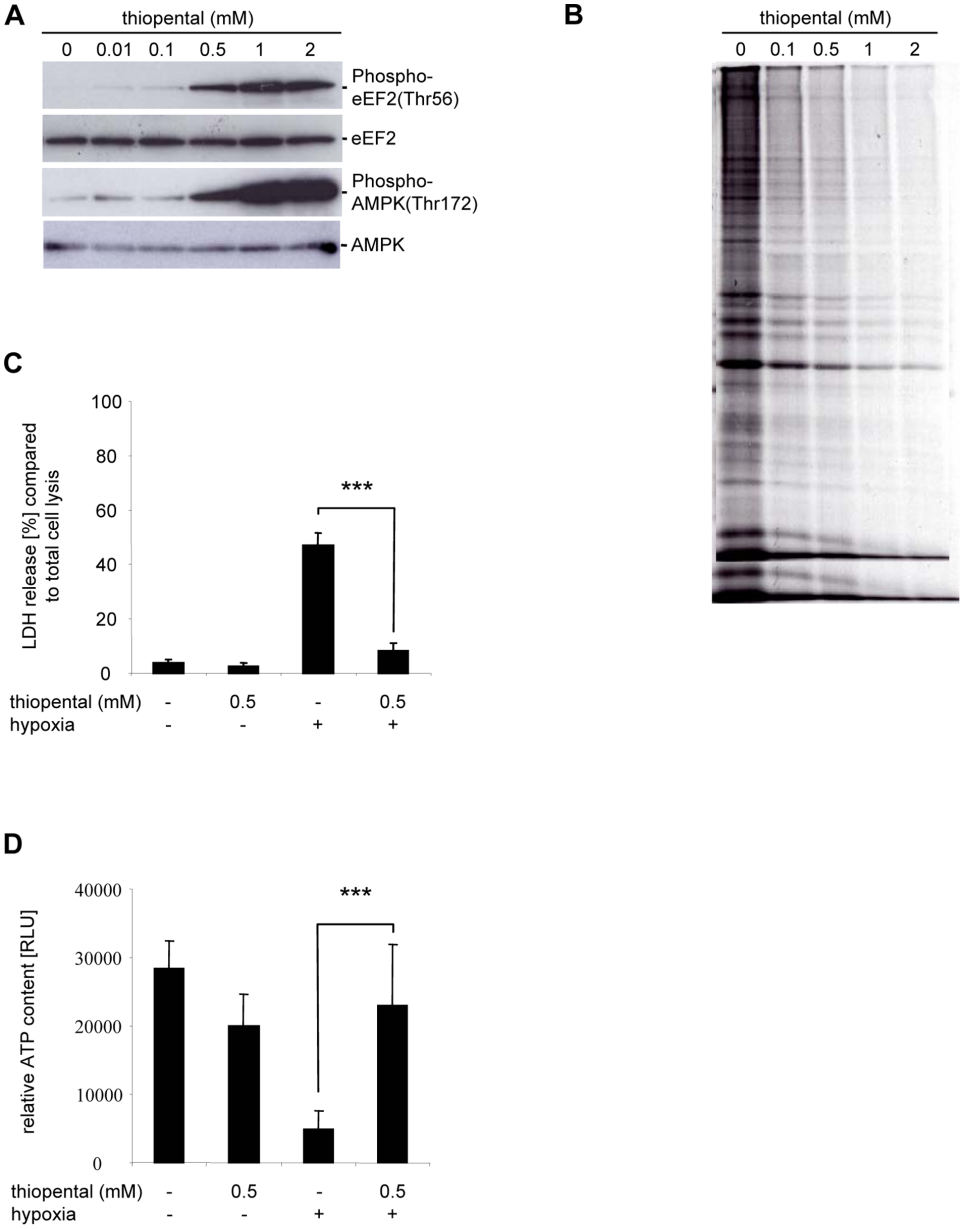
Thiopental inhibits protein synthesis, ameliorates hypoxic damage, and maintains energy balance during oxygen deprivation in primary cortical neurons. In (A), cortical neurons were treated with 10 µM −2 mM thiopental for 30 min and analyzed for phosphorylation of eEF2 and AMPK by immunoblotting. In (B), cortical neurons were left untreated or were exposed to 0.1–2 mM thiopental for 6 h and then pulse labeled with 200 µCi of [^35^S]methionine for an additional 2 h. Cellular lysates were separated by 10% SDS-PAGE and the amounts of newly synthesized proteins were detected by autoradiography on dried electrophoresis gels. In (C/D), cortical neurons were exposed to hypoxia for 48 h in the presence or absence of 0.5 mM thiopental. Cellular damage was determined by an LDH-release assay (C). The relative intracellular ATP-content was measured by an ATP-driven luciferase assay (D). Values represent the mean ± standard deviations of three independent experiments. Statistical evaluation of experimental groups was performed by one-way ANOVA followed by the Bonferroni’s *post hoc* test. The statistically significant difference of oxygen-deprived cortical neurons in the presence or absence of thiopental is shown (***, p<0.001).

The metabolism of differentiated primary cortical neurons and the established SK-N-SH cell line might significantly vary. To analyze the effect of thiopental on translational regulation in terminally differentiated cells, primary neurons were isolated from the cortex of mice and cell cultures were tested for phosphorylation of eEF2 and AMPK. As shown in Figure 9A, thiopental induced a marked phosphorylation of eEF2 and AMPK as previously observed in SK-N-SH cells (Figures 1A, 4A). Furthermore, phosphorylation of eEF2 and AMPK correlated with translational inhibition as demonstrated by metabolic labeling of cortical neurons using [^35^S]methionine (Figure 9B).

In analogy to SK-N-SH cells, the inactivating modification of eEF2 and translational repression in differentiated neurons by thiopental might result in the preservation of intracellular energy content and consequently in protection from hypoxic damage. As shown in Figure 9C, primary cortical neurons cultured in the absence of oxygen displayed a strong LDH release, indicating severe cellular damage by hypoxia. However, in the presence of thiopental damage of cortical neurons by oxygen deprivation was significantly ameliorated. In addition to a reduction of hypoxic damage, thiopental also prevented the depletion of ATP in the absence of oxygen (Figure 9D). In conclusion these results confirm that conservation of cellular energy by inhibition of global protein synthesis and its reallocation to essential ATP-consuming processes may be the key to survive decreased oxygen levels as ATP-supply becomes limiting.

## Discussion

Deprivation of high energy phosphate metabolites during ischemia results in the disruption of ion homeostasis, membrane depolarization, and neuronal cell death [2], [3]. Pharmacologic maintenance of intracellular energy content is therefore a tempting approach to reduce secondary brain injury after ischemic or traumatic damage [55]–[57]. In our study we show that thiopental inhibits global protein synthesis in neurons by mediating the phosphorylation and inactivation of eEF2 and thus reduces ATP consumption and cellular damage in oxygen deprived cells.

Neuroprotection by thiopental has been described in the clinic and the associated reduction of metabolic demands is well established [44], [45], [54]. However, the molecular mechanisms responsible for these effects are largely unknown. Phosphorylation of eEF2 at Thr56 is sufficient to inhibit the energy consuming translational elongation [15] and thus might explain thiopental mediated repression of metabolic demands. eEF2 is a translocase necessary for the movement of the mRNA along the ribosome that in conjunction with aminoacetylation uses most of the ATP/GTP required for protein synthesis [12], [13]. Phosphorylation of eEF2 is mediated by eEF2K [13], a kinase that confers cellular survival by blocking translational elongation and preserving energy balance when supply of the tissue is limited [58]. Ischemic or hemorrhagic stroke results in acute oxygen and nutrient deprivation, depletion of cellular energy content, and neuronal cell death. Therefore, therapeutic induction of eEF2K by thiopental might provide a tool to mediate resistance to these deleterious conditions. Accordingly, pharmacologic inhibition of eEF2K restored sensitivity to hypoxic damage in thiopental-treated neuronal cells.

In our experiments thiopental elevated cytoplasmatic calcium levels and camstatin, a peptide antagonist of calmodulin, prevented eEF2 phosphorylation indicating that this mechanism is essential for thiopental-mediated translational arrest. Calcium in resting cells is about 0.1 µM and the observed 4 to 6 fold increase may be close to optimum for induction of eEF2K or other proteins [59].

Barbiturates attenuate NMDA receptor mediated intracellular calcium elevations and calcium induced membrane depolarisation, suggesting a reduction of excitotoxic neuronal injury [38], [39]. Opposed to these data we observed a moderate increase in intracellular calcium upon thiopental treatment due to extracellular influx. NMDA receptors are most likely not involved because they are non functional in SK-N-SH cells and NMDA treatment does not result in neurotoxicity in these cells [37], [60]. The physiological consequence of the observed cytoplasmatic calcium upregulation is unclear. Anderson et al. described a biphasic effect of barbiturates – low concentrations of barbiturates potentiated NMDA-induced calcium dependent neuronal death, whereas barbiturates at millimolar concentrations were described to be protective and attenuated the NMDA-induced calcium increase [61]. Consistent with Anderson et al. we observed elevated cytoplasmatic calcium levels, but these were accompanied with reduced hypoxic cell death arguing for cytoprotection by thiopental. Therefore, we believe that moderate calcium elevations by thiopental may induce neuroprotective enzymes that antagonize cell death when oxygen availability is limited.

A mechanism which may synergistically lead to thiopental-dependent eEF2 phosphorylation is the induction of AMPK during energy starvation. Barbiturates depress mitochondrial respiration [51], but as cellular ATP-levels are not appreciably reduced during basal metabolic demands this observation was considered as biologically insignificant. However, our results indicate that thiopental stimulates AMPK and eEF2-dependent translational repression, rescues hypoxic neuronal cells from irreversible damage, and thus supports the hypothesis that barbiturate-mediated effects on cellular energy metabolism may become physiologically relevant under conditions of energy deprivation. Although AMPK and eEF2 phosphorylation followed similar kinetics, the consequences of AMPK activation must be considered carefully because the AMPK inhibitor compound c only partially inhibited eEF2 phosphorylation, indicating that this mechanism is not exclusively responsible for thiopental-mediated translational arrest and additional mechanisms may alternatively phosphorylate eEF2 when AMPK is blocked. Calcium/calmodulin dependent activation of eEF2K is independent of AMPK [13], indicating that AMPK in conjunction with calcium/calmodulin are both essential for translational arrest.

AMPK activation is involved in numerous cytoprotective molecular mechanisms such as stress resistance, autophagy, inhibition of plaque formation in Alzheimer’s disease, and energy intake and expenditure [23]–31. All of these effects might contribute to neuroprotection. Indeed, activation of AMPK has been directly associated with salvage of hypoxic neurons [24]. Additionally, AMPK has been associated with induction of eNOS [32], an enzyme that promotes vasodilatation, increases regional blood flow in ischemic brain tissue, and reduces infarct volume [33]. These effects may also contribute to improved oxygenation and a redistribution of cerebral blood flow towards zones of ischemic brain tissue in humans.

Previously we reported that thiopental-mediated modulation of intracellular signal transduction depends on the sulfur substitution at the C2 and thus is a characteristic of thiobarbiturates [37], [62], [63]. In our present experiments we also noted that the oxy-derivate of thiopental, pentobarbital, displayed a significantly reduced ability to induce phosphorylation of eEF2, translational repression and protection from ischemic cell death (Matjaz Humar, unpublished data). These findings are consistent with studies conducted in laboratory animals [64], [65], suggesting that the neuroprotective capacity of thiopental is superior to pentobarbital. In some of these studies neuroprotection by thiopental was associated with reduced radical formation, lipid peroxidation, and inflammation [40]–[42]. Indeed, thiopental scavenges free radicals and acts as a transcriptional repressor of proinflammatory cytokine expression [42], [63]. Interestingly, the formation of neurotoxic mediators in brain lesions is at least partially based on *de novo* protein synthesis including iNOS, COX-2, MMPs or proinflammatory cytokines [4]–[6]. How far translational inhibition of these mediators contributes to thiopental-mediated neuroprotection is currently under investigation.

Furthermore, inhibition of protein synthesis might be useful in the treatment of diseases associated with excessive or deregulated protein expression. AMPK-dependent pathways are crucial in metabolic and structural remodeling, inflammation, cell growth, and tumor suppression [23]–[32]. We observed that chemically related heterocyclic thiourylenes affected protein synthesis comparable to thiopental (Matjaz Humar, unpublished data). Thus heterocyclic thiourylenes might become promising candidates for the treatment of fibrosis, hypertrophy, inflammation, pathological remodeling, or malignant transformation.

Previously we described that thiopental inhibits calcium-dependent caspase activation and apoptosis in neuronal cells [37]. However, our current results clearly demonstrate that hypoxic neuronal cell death is independent of caspase-3. There is clear evidence that cells triggered to undergo apoptosis die by necrosis when energy levels are rapidly compromised [66]. Consistently, oxygen-deprived neurons displayed a dramatic decrease in intracellular ATP that was prevented by treatment with thiopental. Although repletion of the extra-mitochondrial ATP pool might restore the ability of cells to undergo apoptosis [66], cells were protected from hypoxic injury in our experiments. Protection from hypoxic cell damage is most probably unrelated to inhibition of recurrent caspase activation because protein synthesis inhibitors such as cycloheximide or actinomycin D also prevented neuronal injury and intrinsic caspase activation is independent of translation.

A crucial question is how translational inhibitors reduce ischemic neuronal damage. There is only limiting data available demonstrating the reduction of deleterious effects in ischemic neurons by translational inhibitors and participating cytoprotective mechanisms are inadequately resolved [56], [67], [68]. Translational inhibition of damage-promoting proteins such as COX-2 or iNOS might be a relevant aspect. Indeed, we observed attenuated iNOS-dependent NO synthesis paralleled by reduced necrosis in oxygen deprived neurons when cycloheximide or thiopental were administered (Matjaz Humar, unpublished data). In addition, preservation of intracellular energy and its redistribution to processes essential for cellular survival might represent a second option for mediating cytoprotection. Translation consumes a significant amount of ATP derived from the respiratory chain [8], and the therapeutic maintenance of intracellular high energy compounds has been proposed as an effective means to prevent hypoxia induced neuronal injury [9]–[11], [69]. Inhibition of translation might induce a redistribution of the intracellular ATP-pool, then available for the restoration of ion homeostasis after membrane depolarisation and cellular repair.

In patients, a therapeutic benefit of thiopental therapy is controversially discussed, mainly because of severe medical complications and the lack of clinical trials providing clear evidence for neuroprotection by this drug. Our study shows molecular evidence that thiopental and other translational inhibitors modify intracellular energy expenditure, thus supporting cellular adaptation to hypoxia and improving survival. These findings raise the possibility that thiobarbiturates may indeed improve clinical outcome in selected patients. Lack of clinical evidence for thiopental-mediated neuroprotection might be due to insufficient control of medical complications or the time frame barbiturates are employed after traumatic brain injury - the opportunity for the drugs to prevent secondary injury may have past due to the rapid kinetics of energy depletion. Our findings therefore suggest the initiation of new trials to revaluate neuroprotection by thiopental under these aspects. Furthermore, our results indicate that these studies shouldn’t be limited to patients with refractory intracranial pressure but should also include other hypoxic insults, such as stroke.

In summary, the presented findings indicate that inhibition of global protein synthesis by thiopental or other general translational inhibitors effectively protects from hypoxic neuronal cell damage by stabilizing high energy metabolite content under oxygen-limited conditions. Based on our results, we suggest that translational inhibition and preservation of intracellular ATP content by thiopental is mediated by a combined activation of AMPK and calcium/calmodulin complexes, resulting in eEF2 phosphorylation and inactivation. Compounds, based on the structure of heterocyclic thiourylene derivates might therefore reveal a pharmacologically relevant scaffold for the development of novel organ-protective compounds. The identification and modification of chemically active sites within the molecule might improve molecular or metabolic defence mechanisms against limited oxygen supply and allow for efficient screening of compound banks for new organ protective agents containing these biologically relevant structures. Furthermore, our findings suggest examining other general translational inhibitors, agents that maintain mitochondrial respiratory activity during hypoxia, and the therapeutic application of high energy molecules to ameliorate ischemic tissue damage.
